# Stimulus Dependence of Barrel Cortex Directional Selectivity

**DOI:** 10.1371/journal.pone.0000137

**Published:** 2006-12-27

**Authors:** Gabriel D. Puccini, Albert Compte, Miguel Maravall

**Affiliations:** Instituto de Neurociencias de Alicante, Universidad Miguel Hernández (UMH) - el Consejo Superior de Investigaciones Científicas (CSIC), Campus de San Juan, Sant Joan d'Alacant, Alicante, Spain; University of Sydney, Australia

## Abstract

Neurons throughout the rat vibrissa somatosensory pathway are sensitive to the angular direction of whisker movement. Could this sensitivity help rats discriminate stimuli? Here we use a simple computational model of cortical neurons to analyze the robustness of directional selectivity. In the model, directional preference emerges from tuning of synaptic conductance amplitude and latency, as in recent experimental findings. We find that directional selectivity during stimulation with random deflection sequences is strongly dependent on the mean deflection frequency: Selectivity is weakened at high frequencies even when each individual deflection evokes strong directional tuning. This variability of directional selectivity is due to generic properties of synaptic integration by the neuronal membrane, and is therefore likely to hold under very general physiological conditions. Our results suggest that directional selectivity depends on stimulus context. It may participate in tasks involving brief whisker contact, such as detection of object position, but is likely to be weakened in tasks involving sustained whisker exploration (e.g., texture discrimination).

## Introduction

The whisker somatosensory system is crucial to rodents' ability to discriminate object location and identity. The elementary features of whisker stimulus representations continue to be a subject of great interest [1]. It has long been known that neurons throughout the pathway are sensitive to whisker motion direction [2]–[8]. Could directional selectivity be a key element of barrel cortex stimulus representations? Recent work by several groups has examined the organization of directional tuning in barrel cortex (BC) and its afferents [8]–[12] and elucidated mechanisms of directional preference in cortical neurons, showing how synaptic responses vary systematically as a function of the direction of whisker deflection [13]–[15].

One contribution to directional selectivity comes from latency tuning of excitatory inputs [14]. Excitatory synaptic potential amplitudes are somewhat broadly tuned (all whisker directions evoking monosynaptic thalamocortical input), although still with a preferred direction, and inhibitory amplitudes have weak tuning. However, excitatory responses to the preferred direction have short latency whereas those to other directions have longer latencies. Conversely, inhibitory responses have uniform latency. The outcome is that substantial excitation precedes inhibition only for a range of directions close to the preferred one, so that stimuli with this direction enjoy a greater (approximately 2 ms-duration) “window of opportunity” to generate spikes. Thus, for temporally isolated whisker deflections, the relative timing of synaptic inputs is a key mechanism shaping directional selectivity, in conjunction with other mechanisms such as amplitude tuning and changes in spike threshold [13].

Studies of BC directional tuning have typically involved whisker deflections that are temporally isolated from the preceding and succeeding deflections. Conversely, whisker motion induced by texture stimuli is extended in time and has a characteristic intermittent, “noisy” structure organized as a succession of “stick-and-slip” events [16], [17]. Periods of relatively low-frequency, slow whisker motion (free whisking, 5–20 Hz) are interspersed with high-frequency events (several hundred Hz): this structure constitutes a texture's kinetic “signature”, is encoded by neurons in the whisker pathway [16] and may be a substrate for discrimination. Rapidly varying stimuli are useful for studying temporal precision and encoding in the whisker pathway [18]; furthermore, artificial, rapidly varying “white noise” stimuli can be used to predict responses to naturalistic texture waveforms [16]. Strikingly, directional tuning of responses to continuous, rapidly varying stimuli in BC (but not in trigeminal ganglion) appears to be comparatively weak [16]: for instance, tuning is bilaterally symmetric, with each direction and its opposite evoking indistinguishable responses. This result is based on multiunit cluster recordings; if applicable to single units, it would lead to a qualitatively different perspective on directional tuning maps and their significance, as it would imply that BC directional tuning might be limited to brief, isolated stimuli.

In the work presented here, we implemented directional tuning of synaptic conductances in a simple integrate-and-fire model to predict how directional selectivity could be affected by stimuli with rapid variations. We found that one stimulus feature (frequency) can strongly affect the representation of another feature (direction) even in highly simplified models, a phenomenon caused by generic properties of neuronal integration. Directional selectivity is thus modulated by the frequency of ongoing motion. Our results suggest that the role of directional selectivity may be prominent in some situations (e.g. discrimination of object location), but is likely to be negligible in others, such as texture discrimination.

## Results

### Model of synaptically based directional selectivity

**Figure 1:**
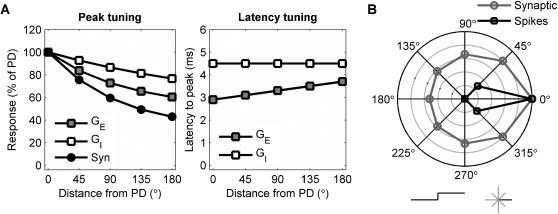
Definition of directionally selective model neuron. **A:** Latency tuning: directional dependence of excitatory and inhibitory synaptic conductance peaks (left) and latencies (right). Symbols: Gray squares, excitatory conductance; white squares, inhibitory conductance; black circles, total synaptic conductance. **B:** Polar plot of directional selectivity of model neuron tested with discrete single deflections in one of eight possible directions (symbolically represented at bottom: solid line is 0° deflection). Responses are on a radial scale normalized to the preferred direction (PD, set to 0°) response (outer circle = 1). Symbols: Circles, gray line: synaptic responses (mean peak evoked synaptic potential per deflection). Squares, black line: spiking responses (mean evoked number of spikes per deflection, counted over a 20 ms window).

To predict how timing-based directional selectivity is expressed during rapidly varying stimuli, we implemented synaptic amplitude and latency tuning in a simple model. An integrate-and-fire neuron received excitatory and inhibitory conductance-based inputs whose amplitude and latency depended on the direction of each “whisker deflection” (Fig. 1A; compare e.g. to Fig. 5 in [14]). We set synaptic conductance durations and time constants for synaptic integration according to experimental data (see Materials and Methods). We first checked responses with temporally isolated single-deflection stimuli in eight directions; as expected, the model matched experimental results, with synaptic directional tuning sharpened by the spike threshold (Fig. 1B).

### Dependence of directional selectivity on stimulus frequency

**Figure 2:**
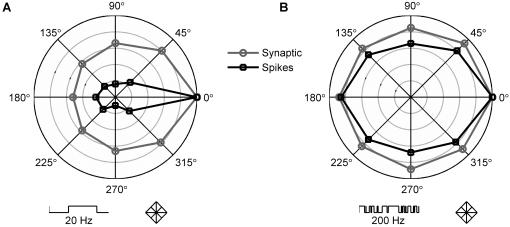
Directional selectivity is sensitive to stimulus frequency. **A:** Plot of directional selectivity tested with 20 Hz random deflection sequence. Deflections occurred at exponentially distributed intervals (symbolically represented at bottom left: each vertical line is a deflection) and were across neighboring positions on a diamond spatial grid (bottom right). Thus, there was a “whisker displacement” from one position to a neighbor every 50 ms on average. Response scales and symbols as in Fig. 1B. **B:** Plot of directional selectivity tested with 200 Hz random deflection sequence. Except for frequency, all other parameters were as for **A** (symbols at bottom represent the faster sequence and the unchanged position grid). Directional selectivity was substantially weakened.

We then generated stimulus sets consisting of sequences of deflections in eight possible directions (described in Materials and Methods). Each such displacement had a well-defined direction, determining activation of excitatory and inhibitory conductances according to the rules of Fig. 1A. Deflection sequences explored a spatial region defined as a grid of discrete positions (Materials and Methods; an example “diamond” grid used in the simulations is shown under the polar plots in Fig. 2). Deflections occurred at exponentially distributed intervals set by a Poisson process, a simple choice of highly irregular stimulus. Directional selectivity at different frequencies was computed by varying the mean inter-deflection interval.

We found that directional selectivity during sustained stimulation with whisker deflection sequences depended strongly on stimulus frequency. For low-frequency deflection sequences (e.g. 20 Hz), directional selectivity maps had a shape similar to that for single deflections or for sequences of isolated deflections (Fig. 2A). However, for higher frequencies (e.g. 200 Hz) directional selectivity was nearly absent (Fig. 2B).

This is because a mechanism based on timing differences must lose selectivity when differences in input arrival times (∼5 ms) are small relative to the synaptic integration time scale. In BC, several estimates of integration time scale are consistent: membrane time constants are ∼10–20 ms; average individual post-synaptic potential duration in vivo is ∼20 ms [15]; the duration of the linear filters determining a neuronal response is ∼20 ms [19]; directly measured integration times range down to ∼1 ms at the beginning of stimulation, but reach ∼20 ms during prolonged or repeated stimulation [20]. At high stimulation frequencies relative to this time scale, successive synaptic inputs evoked by deflections in different directions are not isolated in time, but summed together by the postsynaptic membrane. Without a precise “window of opportunity”, spike generation depends on synaptic summation. Responses to different directions are thus effectively averaged together, and angular preferences will be lost during postsynaptic integration.

Tuning was weakened at high frequencies regardless of the geometry of the whisker position grid used (see below and Materials and Methods). Interestingly, high-frequency tuning plots (Fig. 2B) could be almost fully bilaterally symmetric, comparable to responses to continuous stimuli measured in anesthetized animals [16]. We emphasize that the strikingly different tuning curves in Figs. 2A and B were achieved without changing any model parameters except stimulation frequency.

### Robustness of the frequency dependence of directional selectivity

Our basic model included several simplifying parameter choices, such as a lack of synaptic dynamics and a specific choice for the duration of the time window over which response magnitudes were computed (see Materials and Methods). We therefore tested how various alterations to the model's design could affect the predicted decrease in directional selectivity under high-frequency stimulation. Our aim was not just to extend the model to a slightly more realistic situation, but to evaluate whether its qualitative predictions could be expected to generalize.

**Figure 3:**
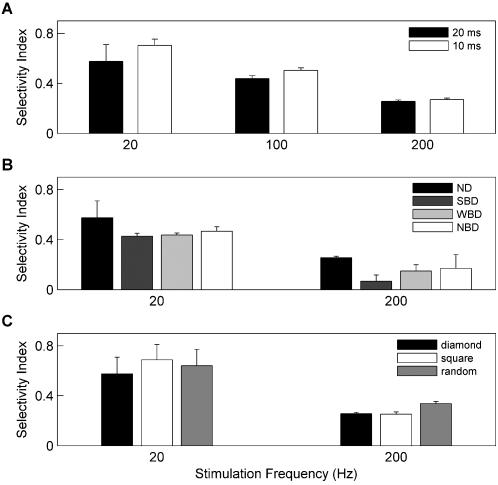
Robustness to changes in key model parameters. All panels represent the “Selectivity Index” (see Materials and Methods) computed on spiking responses. **A:** Dependence on time window for response estimation. Selectivity index as a function of stimulus frequency assessed using two different integration time windows after each deflection (10 and 20 ms). **B:** Dependence on short-term synaptic plasticity. The selectivity index was evaluated for conditions differing in how synaptic depression properties were matched across excitatory and inhibitory conductances (ND: no depression, same model as in other figures; SBD, WBD and NBD defined in Results). **C:** Dependence on choice of grid geometry on which whisker deflections were defined (square: deflections on square grid; diamond: on diamond grid; random: random walk deflections; see Materials and Methods for full explanation and Fig. 2, bottom, for schematic of diamond grid). In all cases, response selectivity was weakened at high frequencies. Error bars: standard error of mean across trials.

First, we evaluated the effects of the choice of time window used to estimate responses and spike probabilities. Although the 20 ms time window used in Figs. 1 and 2 was chosen on grounds of physiological relevance (see Materials and Methods), BC integration time windows can vary – they are layer-dependent [21] and are under the ongoing control of synaptic inhibition [20]. Using a shorter time window to estimate responses would decrease the time over which synaptic inputs were effectively averaged together in our analysis, and could therefore be expected to sharpen the observed directional selectivity. Using a 10 ms time window indeed resulted in sharpened selectivity but did not affect the qualitative result: selectivity was profoundly reduced during high-frequency stimulation (Fig. 3A).

Next, we added short-term dynamics to both excitatory and inhibitory synaptic inputs to the model neuron. Several forms of synaptic depression were assumed, for consistency with recent studies of BC short-term synaptic plasticity that have found different results according to experimental design. Thus, depressing excitatory and inhibitory synapses were either “Strongly balanced” (SBD: both the steady-state value and the relaxation dynamics of depression are the same for excitatory and inhibitory synapses [22]), “Weakly balanced” (WBD: the relaxation dynamics of synaptic depression at 10 Hz stimulation frequency is the same for excitatory and inhibitory synapses, but not the steady-state depressed synaptic efficacy), or “Non-balanced” (NBD: for all stimulation frequencies both the steady state and relaxation dynamics of synaptic efficacies differ for excitatory and inhibitory pathways [20]). Although directional selectivity was somewhat affected by assumptions regarding the detailed form of synaptic dynamics, the central result held in every case: selectivity decreased sharply under high-frequency stimulation (Fig. 3B).

Finally, we compared directional selectivity for deflections on a “diamond” grid to selectivity for deflections on other grid geometries (Fig. 3C; see Materials and Methods for a discussion of grid design). Irrespective of grid choice, directional selectivity dropped off strongly with increasing frequency.

In conclusion, directional selectivity based on smooth tuning of synaptic amplitude and latency is sensitive to stimulus temporal structure. It functions robustly for temporally isolated stimuli and for low-frequency irregular stimulation and breaks down at stimulus frequencies that are high relative to neuronal integration times. This fragility of directional selectivity applies over a wide range of model parameters.

## Discussion

### Robust loss of directional selectivity for high-frequency stimuli

Using a very simple model that incorporates generic features of synaptic integration, we have found that the directional selectivity of BC neurons can be highly dependent on other stimulus features. Varying the stimulus frequency resulted in strikingly different directional tuning, as exemplified by the curves in Figs. 2A and B, which were obtained with identical model parameters. The model was able to account for apparent discrepancies in the literature between the magnitude of directional selectivity to single deflections (e.g. [12]–[15]) and to rapidly varying motion [16].

Stimulus waveforms used in our simulations consisted of sequences of discrete instantaneous displacements, a choice made in the interest of greater simplicity and transparency. It is important to note that to the extent that directional selectivity depends on latency tuning, this choice of stimulus will be biased in favor of directional selectivity. This is because directional tuning based on precise synaptic latency relies on the synchronous activation of afferent neurons. In our model, discrete deflections evoked synaptic conductance waveforms with precise latency, corresponding to a high degree of synchrony. Experimentally, thalamic synchrony is stimulus-dependent [10], [11], [23] and is uniquely high for responses evoked by discrete stimuli with a fast onset (the situation contemplated in our simulations), as compared to other stimuli encountered by the system. For example, synchrony under stimulation with long-duration, continuous waveforms is weaker than under the conditions simulated here [15]. Directional selectivity would therefore be expected to decrease with those stimuli, although the comparison has not been directly made experimentally. Systematic comparisons of directional selectivity for stimuli with variable sharpness or velocity have only been made for isolated ramp-and-hold stimuli [11].

Several lines of experimental evidence indicate that aside from synaptic latency tuning, other mechanisms participate in cortical directional selectivity [11], [13]. Importantly, the degradation of directional selectivity at high frequencies in our model stems from temporal filtering intrinsic to synaptic integration at the membrane – a general, basic neuronal property. We therefore expect this degradation to be a general phenomenon, applicable to directional selectivity mediated by almost any mechanism, including most forms of magnitude tuning as well as spike threshold modulation [13]. Note however that smooth tuning is more susceptible to the averaging effect than all-or-none tuning: neurons receiving all their input from afferents with identical directional tuning can maintain a strong directional preference at much higher frequencies. In sum, loss of directional selectivity under ongoing, continuous, or high-frequency stimuli is likely to be a robust property of BC responses. The influence of stimulus frequency on directional selectivity is an experimentally testable prediction.

### Relevance to tactile coding

Our results suggest that BC directional tuning is at its peak early in a response and for temporally isolated stimuli with a sudden onset: for example, when a whisker first encounters an object. What could be the functional role of this selectivity? Free whisking occurs at frequencies between 5–20 Hz, a range over which directional selectivity is relatively strong. Upon first whisker contact with an object, directional information could thus help the cortex generate a percept of object position and orientation [24]. Later on during exploration of a texture, as whiskers experience high-frequency “stick-and-slip” events separated by short intervals [16], [17], directional information is likely to be “integrated away”. This is consistent with the idea that texture discrimination should be invariant to the texture's position and orientation relative to the whiskers, much as our ability to identify a surface does not depend on how we are running our fingers along it. Ultimately, of course, whether directional selectivity plays a role in whisker stimulus encoding must be settled by directly testing whether rodents can use the direction of vibrissa motion as a sensory cue at different moments during tactile exploration.

Much recent work in the barrel cortex and other sensory cortical areas has been concerned with response modulation by motor inputs, particularly in active sensing, as well as with modulation by states of overall brain activity, for instance by wakefulness or attention (e.g. [1], [25]–[33]). Our results are a reminder that even before considering active modulatory phenomena, response tuning to particular features can be modulated by other stimulus features. The relevance of receptive field properties should be judged in the context of how rats use their whiskers, and what they can feel.

## Materials and Methods

### Model neuron

We implemented a single-compartment leaky integrate-and-fire model neuron [34] with a resting potential of −69 mV, a membrane capacitance of 0.36 µF/cm^2^ (as in experiments in barrel cortex [20], [22]), and a leak conductance of 0.03 mS/cm^2^ (yielding a membrane time constant of 12 ms, consistent with experiments in barrel cortex [20], [22]). The neuron received synaptic inputs in the form of conductance changes with reversal potentials of 0 and −85 mV for excitatory and inhibitory synapses, respectively. The corresponding maximal conductances were modulated according to deflection direction (as indicated in Fig. 1A) and took values of 0.014 mS/cm^2^ and 0.020 mS/cm^2^, respectively, at the preferred direction (PD). The time course of conductance opening was modeled as the difference of two exponentials (with time constants τ_1_ and τ_2_) with variable delayed onset Δ. Latencies to peak were given by Δ+τ_1_τ_2_/(τ_1_−τ_2_)ln(τ_1_/τ_2_), as plotted in Fig. 1A. For excitatory conductances τ_1_ = 3 ms, τ_2_ = 2 ms, and Δ = 0.5–1.4 ms. For inhibitory conductances τ_1_ = 4 ms, τ_2_ = 3 ms, and Δ = 1 ms. This choice of time constants gave time courses consistent with experimental measures of post-synaptic potentials elicited by whisker deflections [14]. When the membrane potential reached the threshold of −60 mV, an action potential was fired and the membrane potential was reset to −70 mV. This value was then held for a refractory period of 2 ms. We verified that the chosen spike threshold sharpened tuning as seen experimentally [13], [15]: this is shown in Figs. 1 and 2.

For simplicity and transparency, the basic model (Figs. 1, 2, and 3A) had no adaptive dynamics (i.e. no synaptic depression or intrinsic spike-frequency adaptation). Synaptic dynamics were implemented in Fig. 3B according to three alternative assumptions: SBD, WBD and NBD, defined by whether depression dynamics and steady states were matched or not across excitatory and inhibitory conductances (see Results). The phenomenological model of synaptic depression was described by two parameters: Γ and τ_D_ [35]. In the SBD case, we used Γ = 0.8, τ_D_ = 400 ms for both excitatory and inhibitory synapses. In the WBD case, we used Γ = 0.8, τ_D_ = 333 ms for excitatory synapses and Γ = 0.6, τ_D_ = 1000 ms for inhibitory synapses. This resulted in a decay time constant for synaptic efficacy of 200 ms at a 10 Hz stimulation rate both for excitatory and for inhibitory conductances. However, steady-state depressed efficacies differed for excitatory and inhibitory pathways. In the NBD case, we used Γ = 0.4, τ_D_ = 200 ms for excitatory synapses and Γ = 0.8, τ_D_ = 2000 ms for inhibitory synapses. For this choice of parameters neither relaxation dynamics nor steady-state efficacy values coincided for excitatory and inhibitory synapses, irrespective of stimulation conditions. Spike thresholds were fixed at −65.5 mV for SBD, −65 mV for WBD and −66 mV for NBD. Our aim was to compare directional selectivity with and without synaptic dynamics, to examine how selectivity could be affected by depression. In plotting Fig. 3B, we therefore focused on steady-state, “adapted” responses, counting spikes starting 300 ms after the onset of stimulation; initial, “non-adapted” responses had the same selectivity as those in the basic model.

Data were averaged over many trials (200 – 2000) consisting of different stimulation sequences drawn from the same statistical distribution (see below).

Simulations were performed in Matlab (The Mathworks, Natick, MA).

### Design of stimulus sequences

We generated stimuli consisting of sequences of successive, discrete “whisker deflections” in eight possible directions. Deflections occurred at exponentially distributed intervals, a simple choice of noisy, rapidly varying stimulus that conforms to Poisson statistics.

Activation of excitatory and inhibitory conductances was fully determined by direction according to Fig. 1A, making it necessary to ensure that each deflection had a well-defined direction. We therefore constrained deflections so that, instead of varying continuously over the *x*-*y* (anteroposterior-dorsoventral) plane of whisking motion, they occurred between discrete positions on one of several possible grids. For example, one could design a square grid on which deflections could be from any one corner to any of the others. In this example, and using cardinal directions for convenience, starting from the lower right-hand (SE) corner deflections could be vertically N (to the upper right-hand corner), horizontally W (to the lower left-hand corner), or diagonally NW (to the upper left-hand corner). Responses to deflections in all eight directions, starting from all corners, were sampled.

Grid choice constrained the stimulus temporal correlation structure: for the square grid example described above, any deflection with an eastward component (E, NE, or SE) was likely to be followed by a deflection with a westward component. In other words, a deflection in one direction had an increased probability of being followed by a deflection in the opposite direction. For low-frequency deflection sequences, responses to each direction were well isolated in time, but for high-frequency sequences, they were “mixed in” (through synaptic integration) with responses to motion with a component in the opposite direction. This could imply a loss of directional selectivity. Note that similar correlations between successive deflections will also arise generally in real whisking movements – for example, whisker protractions are necessarily followed by retractions, and perturbations induced by textures consist of back-and-forth vibrations at frequencies up to several hundred Hz [16].

To avoid conditioning our results to the temporal correlations imposed by any particular grid geometry, we designed several choices of geometry. The principal criteria used in these designs were to ensure adequate sampling of responses to all eight possible directions, while restricting all deflections to have approximately the same amplitude. Approximate equality of deflection amplitudes allowed us to neglect response dependence on any motion parameters other than direction and frequency. Choices of grid geometry included the following: (1) Squares as described above. (2) Squares or diamonds with vertices at the corners, at the midpoints of edges and at the grid center, with deflections allowed between every pair of neighboring vertices. For brevity we refer to this geometry as “diamond” grid; see the lower part of Fig. 2A and B for a schematic. (3) Free random walks, such that each deflection started and ended at a random position. The square grid had the strongest temporal correlations, the random walk had no correlations, and the diamond grid was intermediate. The data shown in Figs. 2, 3A and 3B were obtained using the diamond geometry, chosen because it imposed less restrictive temporal correlations than the square grid and had a more physiologically realistic structure than the random walk: while whiskers are fixed at their base and describe motion that is continuous and close to cyclical, each random-walk deflection started from a random position independently of where the last deflection had ended. Fig. 3C shows a comparison of results for these choices of grid.

### Assessment of responses

Spiking probabilities were measured over a 20 ms time window following each deflection. This coincides with the approximate size of time windows for synaptic integration (see Results) and is the experimentally determined period over which stimulus features influence spiking probability in vivo, as assessed directly from the width of BC spike-triggered stimulus linear filters [19] and the width of the windows relevant to spiking probability prediction under white noise stimuli [16]. We also tested a 10 ms time window (Fig. 3).

Effects of various modifications to the model were compared by computing a “Selectivity Index” that measured the relative magnitude of the response to the preferred direction: Selectivity Index = (response to preferred direction−average of all other responses)/(response to preferred direction). The index was equal to 0 if there was no directional selectivity and equal to 1 if the non-preferred responses were 0% of the preferred response. Fig. 3 represents the Selectivity Index calculated for spiking responses.
